# Effects of Tooth Desensitizers on *Streptococcus mutans* Biofilm Formation Using a Modified Robbins Device Flow Cell System

**DOI:** 10.3390/ijms251910703

**Published:** 2024-10-04

**Authors:** Niraya Kornsombut, Shoji Takenaka, Jutharat Manuschai, Maki Sotozono, Ryoko Nagata, Takako Ida, Risako Sato, Rui Saito, Ryouhei Takahashi, Daichi Sato, Yuichiro Noiri

**Affiliations:** 1 Department of Cariology, Operative Dentistry and Endodontics, Faculty of Dentistry, Graduate School of Medical and Dental Sciences, Niigata University, Niigata 951-8514, Japan; nirayak@nu.ac.th (N.K.);; 2 Department of Restorative Dentistry, Faculty of Dentistry, Naresuan University, Phitsanulok 65000, Thailand

**Keywords:** biofilms, dentin desensitizer, silver diamine fluoride, sodium fluoride, *Streptococcus mutans*, tricalcium phosphate

## Abstract

This study aimed to assess the antibiofilm effects of dentin desensitizers using a modified Robbins device flow cell system. The test desensitizers were Saforide, Caredyne Shield, and Clinpro White Varnish. Standardized dentin specimens were prepared from human single-rooted premolars, treated with one of the materials, and mounted on the modified Robbins device flow cell system. *Streptococcus mutans* biofilms were developed for 24 h at 37 °C under anaerobic conditions. Scanning electron microscopy, fluorescence confocal laser scanning microscopy, viable and total cell counts, acid production, and gene expression analyses were performed. A wavelength-dispersive X-ray spectroscopy electron probe microanalyzer was used to analyze the ion incorporations. Clinpro White Varnish showed the greatest inhibition, suggesting its suppression of bacterial adherence and transcription of genes related to biofilm formation. Saforide reduced only the number of viable bacteria, but other results showed no significant difference. The antibiofilm effects of Caredyne Shield were limited. The uptake of ions released from a material into dentin varies depending on the element. Clinpro White Varnish is effective for the short-term treatment of tooth sensitivity due to dentin demineralization. It prioritizes remineralization by supplying calcium and fluoride ions while resisting biofilm formation.

## 1. Introduction

Dentin hypersensitivity occurs when the dentin surface is exposed and dentin tubules are accessible from the pulp to the oral environment. This exposure can be caused by various factors, including gingival recession, attrition, and erosive lesions [1,2]. The prevalence of dentin hypersensitivity ranges from 3% to 98%; it is slightly higher for women and peaks at 30–40 years [3]. The accumulation of bacterial biofilm on exposed dentin surfaces can lead to pathological alterations in the pulpal tissue. This process is driven by the bacterial invasion of the dentinal tubules, the diffusion of metabolites, and inflammatory reactions. Studies have shown that bacterial infiltration of human dentin can occur and affect fluid filtration within dentin tubules [4]. For cases of carious dentin, reactions have been observed in the dentinal tubules beneath the translucent zones, indicating the presence of salivary, serum, and microbial components [5]. Acid metabolites from *Streptococcus mutans* can damage deeper dentin tissues and infiltrate the dentinal tubules towards the pulp. This causes inflammation that may lead to pulpitis and necrosis [6,7,8,9].

In recent years, tooth desensitizers containing bioactive ingredients that suppress tooth sensitivity and are expected to remineralize teeth have been developed. The uptake of various ions by tooth structures with reduced acid resistance may help inhibit the progression of dental caries and biofilm formation. Bacterial adhesion is influenced by the surface characteristics of the tooth such as the topography, roughness, hydrophobicity, and electric charge [10,11]. The incorporation of metal ions into the target material improves anti-biofilm activity [10,11]. For example, fluoride has been known for its bactericidal effect via the disruption of metabolism because of the inhibition of bacterial enzymes and membrane function [10]. However, knowledge regarding their ability to inhibit biofilm formation on exposed dentin is limited. In addition, biofilm formation for up to 24 h after application may affect ion uptake, although the release of active ions is highest immediately after application.

Thus, we evaluated the antibiofilm effects of three commercial dental desensitizers in this study: Clinpro White Varnish (CP; 3M Dental), Caredyne Shield (CD; GC Corporation), and Saforide (SDF; Bee Brand Medico Dental). Ion incorporation with dentin in the presence of the biofilm was observed. The active ions included in these products react with the tooth structure and crystallize and physically seal the dentinal tubules. CP is a fluoride varnish widely used to relieve dentin hypersensitivity. CD is another dental desensitizer that contains zinc. SDF is a silver diamine fluoride solution known for its dual role in preventing dental caries and providing desensitization. CP is a 5% sodium fluoride (NaF) solution containing tricalcium phosphate (TCP) and fluoride ions. A previous study evaluating different methods of applying fluoride varnish, including CP, showed that indirect remineralization leads to significantly higher Vickers microhardness than direct remineralization, indicating the reliability of CP for remineralization [12]. Furthermore, CP has been recognized as an effective treatment for dentin hypersensitivity. The therapeutic efficacy of this treatment in lowering sensitivity symptoms has been demonstrated [13].

CD, a desensitizer containing fluorozinc silicate glass, was designed to treat dentin hypersensitivity by occluding exposed dentinal tubules and providing a protective barrier against external stimuli. Zinc ions have demonstrated various beneficial effects, including reducing enamel and dentin demineralization and inhibiting plaque growth, biofilm formation, and dentin collagen degradation [14,15,16,17]. Several studies have evaluated the efficacy of CD for dentin hypersensitivity. A pilot trial comparing CD and Nanoseal, a widely used desensitizer in Japan, found CD to be effective in treating cervical dentin hypersensitivity [17,18]. Another study assessed the inhibition of dentin demineralization using CD. The results indicate that CD effectively inhibits demineralization, further supporting its use as a desensitizer and caries inhibitor [19].

Based on previous reports, SDF is a promising desensitizing agent for treating dentin hypersensitivity. SDF is a 38% silver diamine fluoride that functions as a desensitizer by creating a squamous layer over the exposed dentin and partially plugging dentinal tubules. This reduces fluid shift in the tubules, which is responsible for tooth sensitivity [20]. The silver and fluoride components of SDF promote tooth desensitization through a series of chemical reactions [20]. SDF has demonstrated high efficacy for caries prevention and arrest and often outperforms other preventive options. Its ease of application, cost-effectiveness, and ability to be used in community settings make it valuable for caries management, particularly for high-risk populations and children [21,22]. Another study indicated that the interaction of SDF with hydroxyapatite leads to the formation of fluorapatite, which is beneficial for preventing dental caries [23]. However, the black staining of carious lesions after SDF application remains a major disadvantage [24,25].

While their desensitizing properties are well documented, their ability to inhibit biofilm formation is less understood. Thus, we aimed to compare the efficacies of the active ingredients of CD, SDF, and CP as biofilm formation inhibitors using a modified Robbins device flow cell system (MRD). The MRD flow cell system is a versatile device that forms biofilm under a controlled fluid share force and facilitates the analyses of various factors such as the localization of bacteria, physiological diversity, the effect of antimicrobial agents, and the interaction between the biofilm and adhesion interface [26,27]. The results obtained using this device are more reliable than other static in vitro models because it reproduces the oral cavity environment to some extent. 

The null hypothesis was that there were no significant differences between the antibiofilm properties of the three commercial dental desensitizers.

## 2. Results

### 2.1. Scanning Electron Microscopy Observation

**Figure 1:**
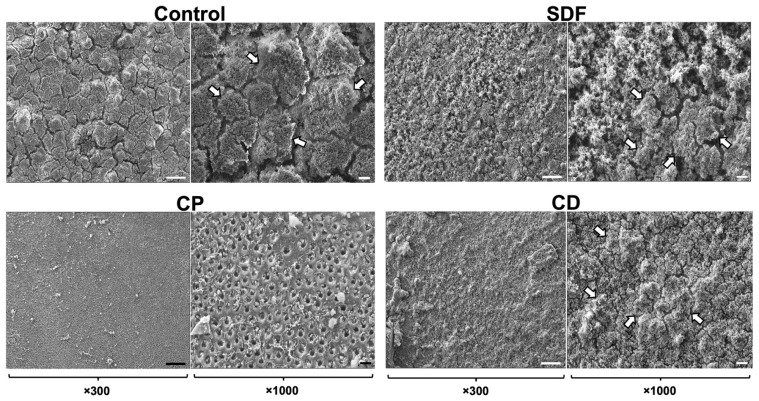
Representative scanning electron microscopy images of *S. mutans* biofilms on each specimen after 24 h of incubation. A significant number of mature biofilm clusters developed throughout the field of view in the control, SDF, and CD groups. An extremely small number of clusters were observed on the dentin surfaces of the CP group. White arrows indicate the biofilm clusters. CP: Clinpro White Varnish; SDF: Saforide; CD: Caredyne Shield. Scale bars = 100 μm (×300) and 20 μm (×1000).

Figure 1 shows the *S. mutans* biofilm formation on the dentin specimens after 24 h of incubation. *S. mutans* biofilms formed successfully on all test materials. A significant number of biofilm clusters developed in the control, SDF, and CD groups across the entire field of view. However, the CP group demonstrated significantly reduced biofilm formation relative to the control, SDF, and CD groups and noticeably sparser and thinner biofilm clusters.

### 2.2. Confocal Laser Scanning Microscopy Observation

**Figure 2:**
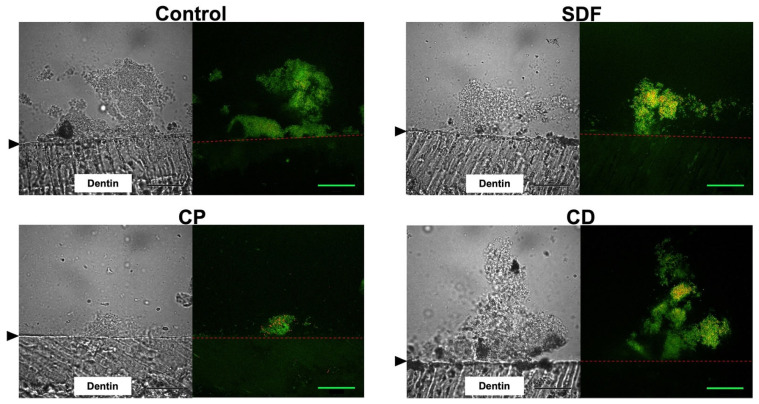
Representative cryosectioned images of *S. mutans* biofilms stained with a Live/Dead staining. Live cells are stained green, while dead cells are stained red. Left: transmission image; Right: fluorescent images. The black arrow and red dashed line indicate the interface between the dentin surface and the biofilm. CP: Clinpro White Varnish; SDF: Saforide; CD: Caredyne Shield; Scale bar = 30 μm.

Cryosectioned images of *S. mutans* biofilms after 24 h of incubation are shown in Figure 2. Using a LIVE/DEAD BacLight Bacterial Viability Kit (Thermo Fisher Scientific, Waltham, MA, USA), live cells in the *S. mutans* biofilms appeared green (SYTO9), while dead cells appeared red (propidium iodide). The confocal laser scanning microscopy (CLSM) images revealed that *S. mutans* biofilms had successfully developed on all materials, and most of the bacteria within the biofilm were alive. However, the biovolume mass of the CP group was significantly lower than those of the control, SDF, and CD groups.

### 2.3. Viable and Total Cell Counts of S. mutans Biofilm

**Figure 3:**
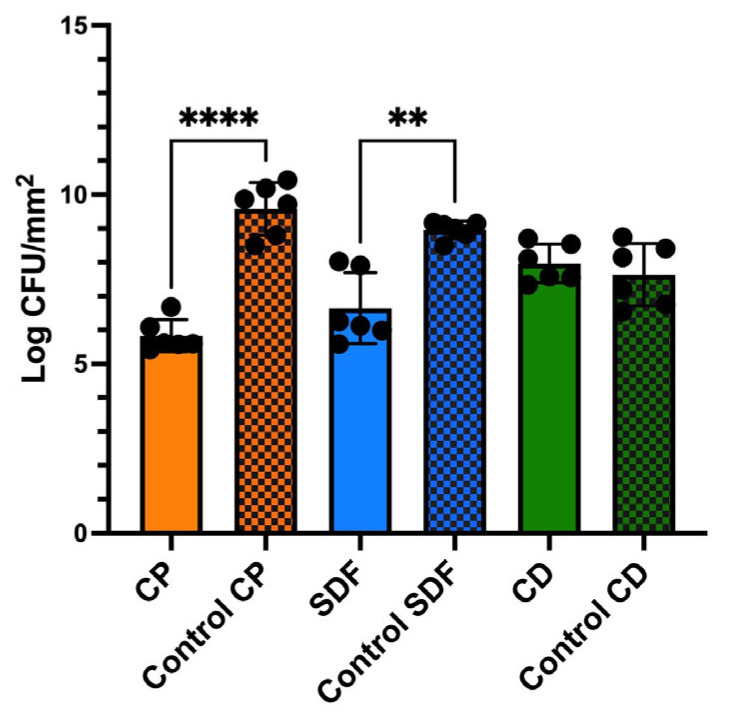
Counts of viable *S. mutans* biofilm cells after 24 h of incubation measured by colony counts. Data are presented as the means ± standard deviation for six replicates (*n* = 6). The colored grid bars represent each experimental group corresponding to its control group. (Kruskal–Wallis, Dunn’s multiple comparison.) Significance levels are indicated as follows: ** *p* < 0.01, **** *p* < 0.0001. CP: Clinpro White Varnish; Control CP: Control group for Clinpro White Varnish; SDF: Saforide; Control SDF: Control group for Saforide; CD: Caredyne Shield; Control CD: Control group for Caredyne Shield.

Figure 3 shows the number of viable cells in the specimens after 24 h of incubation. The CP group had the fewest viable cells, which decreased to 1/1000 of that of the control group (*p* < 0.0001). The SDF group had 1/100 more viable cells than the control group (*p* < 0.01). The viable cell counts of the CD and control groups were not significantly different.

**Figure 4:**
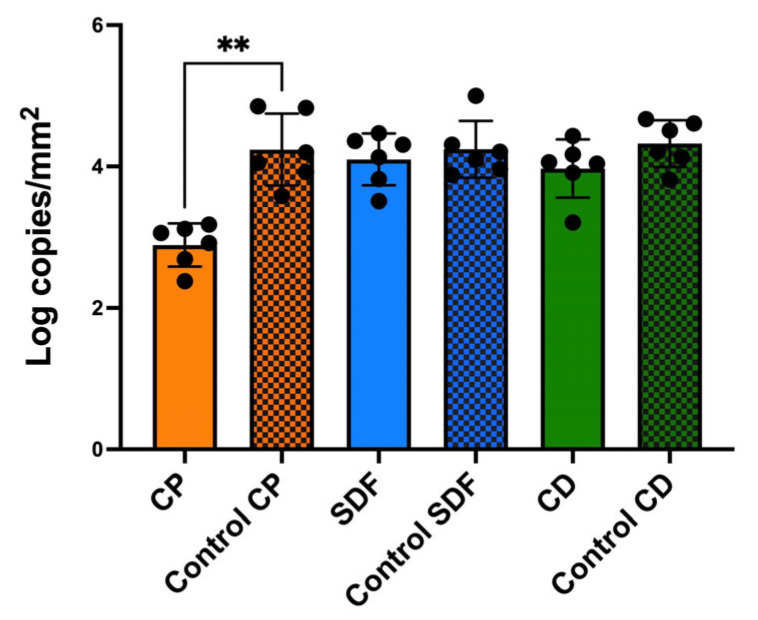
The total bacterial counts of *S. mutans* biofilms formed on CP, SDF, and CD after 24 h of incubation. Results are presented as means ± standard deviations for six replicates (*n* = 6). The colored grid bars represent the control group for each experimental group. (** *p* < 0.01, Kruskal–Wallis, Dunn’s multiple comparison). CP: Clinpro White Varnish; Control CP: Control group for Clinpro White Varnish; SDF: Saforide; Control SDF: Control group for Saforide; CD: Caredyne Shield; Control CD: Control group for Caredyne Shield.

The CP group had a significantly lower total bacterial cell count (*p* < 0.01) than the control group (Figure 4). However, the other experimental groups did not show significantly different cell counts.

### 2.4. Relative Adenosine Triphosphate Bioluminescence Content

**Figure 5:**
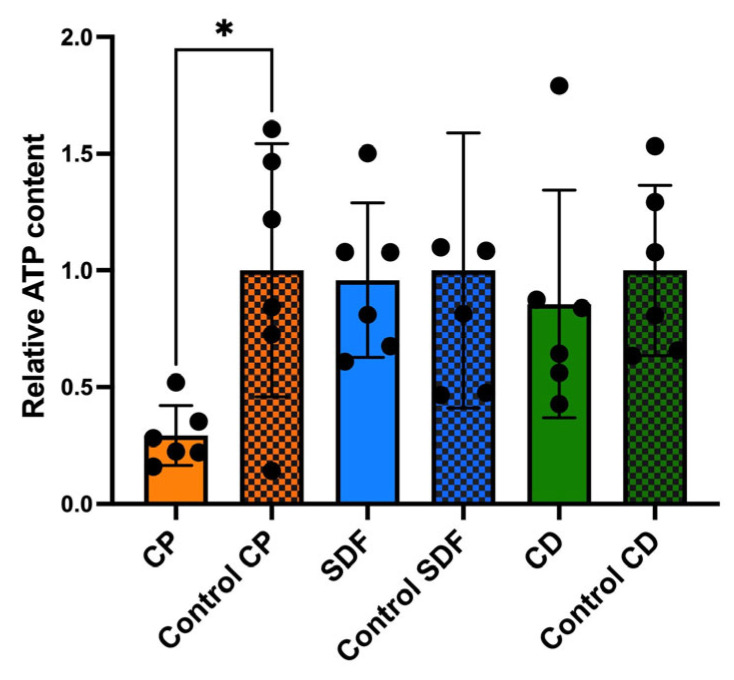
The relative adenosine triphosphate bioluminescence assay measures the content of residual bacterial cells on the specimen surface after the biofilm detachment procedure. The results are presented as the means ± SD for six replicates (*n* = 6). (* *p* < 0.05, Kruskal–Wallis, Dunn’s multiple comparison). CP: Clinpro White Varnish; Control CP: Control group for Clinpro White Varnish; SDF: Saforide; Control SDF: Control group for Saforide; CD: Caredyne Shield; Control CD: Control group for Caredyne Shield.

After 24 h of incubation, the adenosine triphosphate (ATP) content of the CP group was significantly lower than that of the control group (Figure 5, *p* < 0.05). However, no significant differences were observed among the other experimental groups.

### 2.5. Acid Production

**Figure 6:**
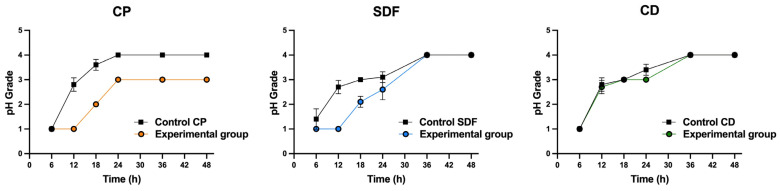
Changes in acid production by biofilm on specimens over 48 h. The pH levels were measured after a specified incubation time using a modified 7-point grading scale with 0.5 intervals based on a 4-point system. A pH of 0 indicates low acid production, while 5 indicates high acid production rates. The results showed no significant difference between the experimental and control groups across all the tested materials (*n* = 5). (Mann–Whitney U) CP: Clinpro White Varnish; Control CP: Control group for Clinpro White Varnish; SDF: Saforide; Control SDF: Control group for Saforide; CD: Caredyne Shield; Control CD: Control group for Caredyne Shield.

Figure 6 shows the acid-producing activities of biofilms on the specimens. The acidification of the medium was not significantly affected by the ions included in the material (*p* > 0.05). Nevertheless, the CP group demonstrated a tendency toward reduced acid production relative to the corresponding control group. The SDF group showed a tendency to delay the acid production of *S. mutans* biofilms for up to 30 h, while the CD group demonstrated acid production rates comparable to those of the control group.

### 2.6. Electron Probe Microanalyzer

**Figure 7:**
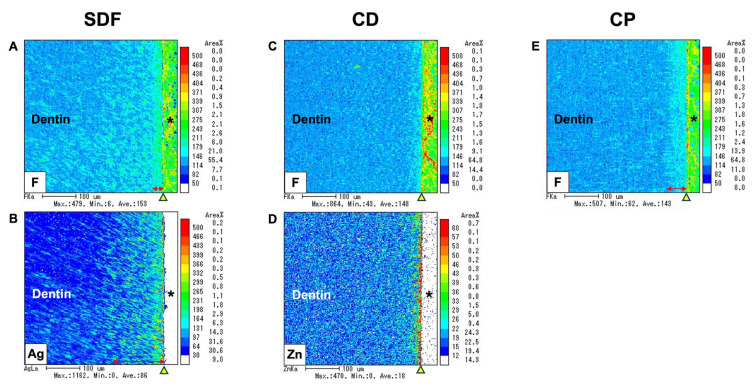
Elemental distribution images using an EPMA. Fluoride (F), silver (Ag), and zinc (Zn) ions were incorporated into the dentin surface after applying SDF (**A**,**B**), CD (**C**,**D**), and CP (**E**). The yellow arrow indicates the interface between the dentin surface and the biofilm. The red arrow indicates the depth of ion incorporation into the dentin specimen. Asterisk (*): biofilm; SDF: Saforide; CD: Caredyne Shield; CP: Clinpro White Varnish.

The elemental distribution images obtained from the electron probe microanalyzer (EPMA) analysis are shown in Figure 7. This analysis revealed that ion incorporation into dentin varied with the depth depending on the specific element. The incorporation of fluoride, silver, and zinc ions into dentin was observed across the experimental groups. Fluoride and silver ions were incorporated for the SDF group, fluoride and zinc ions were incorporated for the CD group, and only fluoride ions were incorporated for the CP group. All experimental groups had fluoride ions incorporated into dentin, although the depth of fluoride ion uptake varied with the materials used; it ranged from 10 to 30 μm. The depth of silver ion incorporation in the SDF group reached approximately 100 μm, while zinc ion incorporation in the CD group was limited to the superficial dentin surface. These findings underscore the material-dependent differences in ion incorporation into the demineralized dentin layer and highlight the varying depths and extents of ion uptake for the different elements and treatment groups.

### 2.7. Gene Expression Analysis

**Figure 8:**
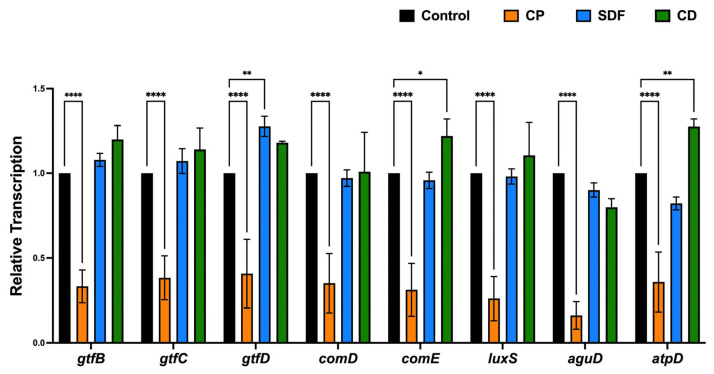
Expression profiles of genes involved in glucosyltransferases, quorum-sensing systems, acid tolerance, and acid production. The results are presented as the mean ± standard deviation for six replicates (*n* = 6). (Kruskal–Wallis, Dunn’s multiple comparison). Significance levels are indicated as follows: * *p* < 0.05, ** *p* < 0.01, **** *p* < 0.0001, for comparison with control groups. CP: Clinpro White Varnish; SDF: Saforide; CD: Caredyne Shield.

The expression profiles of genes encoding glucosyltransferases (*gtfB*, *gtfC*, *gtfD*); quorum-sensing systems (*comD*, *comE*, *luxS*); acid tolerance (*aguD*); and acid production (*atpD*) are shown in Figure 8. For the CP group, the transcription of all genes tested in this study was significantly lower than that for the control group (*p* < 0.0001). However, there was no significant downregulation of gene expression in the SDF and CD groups relative to the control group. Conversely, the CD group showed a significantly upregulated transcription of *gtfD*, *comE*, and *atpD*, while the SDF group showed a significantly upregulated transcription of *gtfD*. In addition, the upregulation was more pronounced in the CD group than in the SDF group.

## 3. Discussion

As dentin desensitizers incorporate various metal elements with fluoride, silver, and zinc ions, they are expected to have antibiofilm properties in addition to their role in reducing dental hypersensitivity. An MRD was used to assess their antibiofilm effects. Various flow environments and the interactions between microorganisms were simulated under incubation for 24 h at 37 °C under anaerobic conditions, and the dentin surfaces were evaluated. The MRD, which closely mimics the natural conditions of the oral cavity, is an advanced tool developed to study dental biofilms under controlled flow conditions. Additionally, the MRD allows researchers to investigate the spatial distribution of viable and non-viable bacteria within biofilms and assess the impact of various treatments on their structure and microbial community composition [27,28]. Hence, the MRD is valuable for dental biofilm research and provides a robust platform for studying biofilm formation, treatment efficacy, and microbial interactions under flow conditions [29,30]. The MRD cannot be used for long-term culture due to the narrow flow path. Therefore, it is suitable for short-term culture, as in this experiment.

The null hypothesis was rejected. The antibiofilm effects of the test materials varied. Among the test material groups, the CP group had the most significantly different viable colony count (the number of living microorganisms in the biofilms on the specimens) relative to the control group, as shown in Figure 3. The total bacterial counts and ATP assay were consistent with the viable cell counts (Figure 4 and Figure 5), indicating that the antibiofilm effect of CP was greater than those of the other materials. Furthermore, morphological observations revealed that the CP group more significantly suppressed biofilm formation than the SDF and CD groups. However, most of the bacteria present in the specimens from all test groups remained viable. This indicated that the ions released from the materials were ineffective in killing all the bacteria in the biofilm. Nevertheless, the biofilm clusters in the CP group were the thinnest, smallest, and sparsest. CP was the most effective in reducing the number of bacteria and the number of biofilm clusters. These findings suggest that CP prevented bacterial adhesion to dentin specimens. Consistent with previous findings, the 5% NaF varnish containing TCP was the most effective in reducing cariogenic bacterial levels (mutans streptococci and lactobacilli), suggesting its superior antibacterial properties in children with severe early childhood caries [31]. Moreover, fluoride treatment inhibited the growth of *S. mutans* biofilms, particularly during the early stages of formation. The antibiofilm and anti-extracellular polysaccharide (EPS) formation activities of brief fluoride treatments increased with the concentration [32]. Our gene expression analysis in the CP group supports this hypothesis. Among the test materials, only CP significantly downregulated the relative transcription of genes associated with glucosyltransferases (*gtfB* and *gtfC*), quorum-sensing systems (*comD*, *comE*, *LuxS*), acid tolerance (*aguD*), and acid production (*atpD*) (Figure 8). The downregulation of genes related to glucosyltransferases reduces glucan production, and the disruption of the quorum-sensing system leads to defective biofilm maturation. The genes of *aguD* and *atpD* are deeply involved in bacterial pathogenicity. By downregulating the expressions of these genes, CP may suppress biofilm formation at the dentin surface and facilitate its detachment. A previous study analyzed the *S. mutans* transcriptome in the presence of NaF and revealed a reduction in overall gene expression levels. The downregulation of genes encoding metabolic transporters and sugar internalization suggests that fluoride can significantly alter bacterial metabolic pathways [33]. We also evaluated the acid-producing activities of biofilms on the specimens (Figure 6). There were no significant differences between the test groups; however, acid production in the CP group was lower than that in the control group. NaF interferes with bacterial metabolic activities, particularly those involved in acid production. A previous study found that NaF inhibited acid production and acid tolerance by disrupting membrane proton permeability and F-ATPase activity. This limits the ability of *S. mutans* to maintain pH homeostasis and produce acids [34]. It inhibits glycolysis and other enzymes involved in bacterial metabolism, which results in changes in the expression of genes related to these metabolic pathways [34,35]. This aligns with the outcome of our gene expression analysis, which indicated that CP can reduce the transcription of genes related to biofilm formation.

The viable cell counts of the SDF group were also significantly different from those of the control group. According to previous studies, SDF shows significant antibacterial effects against *S. mutans* biofilms, which contributes to its effectiveness as a caries-arresting agent [36,37]. However, there were no significant differences in the total cell counts and ATP assays of the SDF and control groups. Morphological observations revealed that the SDF group did not demonstrate the suppression of biofilm formation relative to the control group. Both the SDF and control groups showed the same significant biofilm clusters. Furthermore, the investigation of gene expression investigation in this study provided evidence that SDF did not significantly decrease the relative transcription of any genes related to biofilm formation. Based on the findings of this study, SDF can kill bacteria in the biofilm, but it is ineffective in preventing bacterial attachment to dentin specimens. These findings suggest that TCP, which has no bactericidal effects, inhibits biofilm formation. In addition, the CD group showed no significant reduction in the number of bacteria or biofilm formation in any test experiment. In contrast with our findings, Kumagai et al. found that CD significantly inhibited biofilm formation by *S. mutans* relative to an acidulated phosphate fluoride (APF) gel, as indicated by reduced bacterial colonies and the absence of biofilm on treated enamel surfaces [38]. This discrepancy may be attributed to the differences in the culture conditions and evaluation methods used for the in vitro experiments.

In this study, we investigated the uptake of fluoride, zinc, and silver ions into dentin using EPMA. All test groups demonstrated fluoride incorporation, and the CD and SDF groups demonstrated zinc and silver incorporation, respectively, due to their compositions. However, the incorporated ions failed to prevent biofilm growth in the CD and SDF groups. Contrary to our findings, an earlier study found that 38% SDF had an antimicrobial effect strong enough to potentially prevent biofilm formation in *S. mutans* [39]. Additionally, a previous in vitro study investigating the effects of zinc and fluoride on remineralization using simulated carious lesions found that zinc may enhance the efficiency of antibiofilm and tooth remineralization [40]. The present findings may be due to the stability of the 38% SDF solutions, which revealed variations in the concentrations of free fluoride and silver ions. Over time, the fluoride content tended to increase, whereas the silver content decreased, which is crucial for clinical applications [41]. From these findings, it can be inferred that the antibacterial mechanism of fluoride ions is not the only factor that plays a significant role in inhibiting *S. mutans* biofilms in the CP group. The results of the current study may be attributed to the inherent properties of the CP material, which supports the fluoride function of the material in counteracting the antibiofilm effect. Additionally, this study employed an alternative methodology that simulates oral conditions using an MRD flow cell, potentially leading to different outcomes. Moreover, the combination of NaF and TCP synergistically enhanced remineralization. TCP was designed to prevent premature fluoride–calcium interactions, which can lead to the precipitation of insoluble calcium fluoride. By encapsulating TCP with organic molecules such as fumaric acid, calcium ions were isolated until they were applied to the tooth surface, where they could constructively interact with fluoride to promote remineralization [42,43]. Recent studies have demonstrated that fluoride varnishes containing TCP effectively inhibit caries progression by enhancing mineral loss protection and providing remineralization potential [44,45]. Furthermore, the combination of TCP and fluoride assisted in the gradual infiltration of dentin by fluoride ions. This enhanced the anti-caries effect relative to conventional fluoride varnish alone. TCP also allows for the deeper infiltration of calcium ions. This contributes to remineralization and forms a protective barrier within dentin tubules, especially demineralized dentin [46]. NaF alone had limited anticariogenic activity over time, but the addition of TCP appeared to have enhanced its effectiveness. Over three months, the TCP fluoride varnish demonstrated superior long-lasting antibacterial activity against MS and LB relative to other varnishes without TCP [31]. This outcome may also corroborate the superior antibiofilm performance of CP compared to that of other materials. 

This study has several limitations. A single-species biofilm model composed of *S. mutans* was used. This model may not fully represent the diverse microbial community of dental biofilms (where multiple species interact) and processes including resistance to antimicrobial agents. However, *S. mutans* is highly acidogenic, and it creates harsh conditions for the dental interface, making it a useful method for comparing the antibiofilm effects of these materials. The study also used a 24 h biofilm model, representing an immature biofilm. The early 24 h of incubation is a critical phase where microbial adhesion and biofilm architecture are established. This incubation period has been set for understanding how desensitizers inhibit initial microbial adhesion. This timeframe is widely accepted and allows for measurable biofilm development [47,48]. However, mature biofilms, which are structurally and functionally different, generally show greater resistance to stressors [32]. Thus, the antibiofilm effects of these materials on mature biofilms may be more limited. Future studies using a multi-species biofilm model with a longer growth period are needed to verify the antibiofilm properties of tooth desensitizers.

By investigating the behavior and characteristics of biofilms under controlled dynamic conditions, this study provides significant insights that are valuable for predicting clinical outcomes. In summary, CP can suppress bacterial adhesion and initial biofilm formation for at least 24 h after application while continuing to supply the calcium necessary for the remineralization of the tooth structure. In dental clinical practice, it is an effective short-term treatment for tooth sensitivity due to dentin demineralization, as it prioritizes remineralization by supplying calcium and fluoride ions while resisting biofilm formation. CD and SDF allowed biofilm formation on their surface, but they provide ions to the dentin while physically preventing acid attacks during the initial 24 h of biofilm formation. The detailed mechanism remains unclear, but the combination of NaF and TCP may have synergistic effects in protecting the dentin from cariogenic damage.

## 4. Materials and Methods

### 4.1. Specimen Preparation

**Figure 9:**
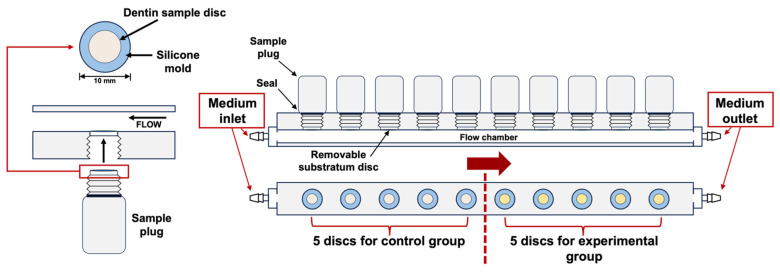
The Modified Robbins device (MRD) used in this study. A pair of dentin specimens were mounted on a sampling plug of the MRD. Five dentin specimens in the sampling plug were allocated to the experimental group, while the other five were allocated to the control group.

Three types of dentin desensitizers were tested in this study: (a) SDF (silver diamine fluoride; Bee Brand Medico Dental, Osaka, Japan), (b) CD (fluorozinc silicate glass; GC Corporation, Tokyo, Japan), and (c) CP (5% NaF with TCP containing fluoride ions; 3M Dental, St. Paul, MN, USA) (Table 1). A pair of dentin specimens (1.5 mm in thickness) were prepared from 75 human premolars using a low-speed diamond saw (Isomet; Buehler, Lake Bluff, IL, USA) under a water coolant and polished using 4000 grit SiC paper (Marumoto Struers KK, Tokyo, Japan). The study protocol was approved by the Niigata University Ethics Committee (approval number 2022-0069). To remove organic tissue, the specimens were immersed in 2.5% sodium hypochlorite (NaOCl) for 1 min, ultrasonicated with 17% EDTA for 1 min, immersed again in 2.5% NaOCl for 1 min, and washed with distilled water. The paired dentin specimens were randomly assigned to three material groups: CP, CD, and SDF. One of the paired specimens was allocated to the experimental group, and the other served as a control. This approach eliminated individual differences in the dentin surface; each experimental specimen had a corresponding control. Ten specimens were attached to the sampling plugs of the MRD using a 10 mm silicone ring (Figure 9). The MRD was sterilized using ethylene oxide gas for 4 h. Five specimens were treated with each dentin desensitizer according to the manufacturer’s instructions. They comprised the experimental group. The other specimens were not treated with any desensitizers and comprised the control group. All specimens were mounted in a flow cell chamber (Convertible Flow Cell^®^ CFCAS0003, IBI Scientific, Dubuque, IA, USA).

### 4.2. Bacteria and Biofilm Formation

*S. mutans* UA159 was cultured as previously described [49]. Before inoculation, the optical density of the bacterial suspension was adjusted to 0.05 at 600 nm. A 20 mL adjusted saliva solution was pumped into a chamber at a flow rate of 2 mL/min and left static for 30 min at 37 °C to allow for the salivary pellicle. The saliva was prepared according to a previously described procedure [49]. Saliva samples were diluted (1:10) with a sterile Ringer solution containing 0.05% cysteine (Sigma-Aldrich, St. Louis, MO, USA). The diluted solution was centrifuged for 10 min at a speed of 10,000× *g* at 4 °C, and the supernatant was filter-sterilized. Next, the same volume of *S. mutans* was pumped into the chamber and left static for 30 min to enable initial adhesion. After 1 h, the flow rate of the medium was maintained at 2 mL/min. The medium used had 1/10 of the strength of BHI broth containing 0.05% sucrose (pH 7.4). The *S. mutans* biofilm was allowed to form for 24 h at 37 °C under anaerobic conditions. Bacterial biofilms were cultivated anaerobically on the dentin surface for 24 h at 37 °C using a continuous flow condition with the MRD. The system included a medium reservoir, a peristaltic pump, and a waste carboy connected via silicone tubing.

### 4.3. Morphological Observation Using Scanning Electron Microscopy

Following the incubation, the specimens were removed from the chamber and rinsed twice with sterile phosphate-buffered saline (PBS) (pH 7.0). The specimens were fixed overnight at 4 °C in 2.5% glutaraldehyde. Subsequently, the specimens were rinsed twice with PBS, dehydrated through an ascending series of ethanol solutions (50–100% *v*/*v*), and sputter-coated with gold-palladium [50]. The biofilms were observed using scanning electron microscopy at magnifications of 300× and 1000×.

### 4.4. Fluorescent Staining and CLSM Observation

After incubation for 24 h, the specimens were stained with a fluorescent bacterial viability kit (LIVE/DEAD BacLight Bacterial Viability Kit; Thermo Fisher Scientific, Waltham, MA, USA) for 30 min at room temperature in the dark [51,52]. The specimens were embedded in 4% carboxymethyl cellulose sodium salt (Section Lab Co., Ltd., Hiroshima, Japan). An adhesive-film method was used to create an 8 μm thick frozen section perpendicular to the enamel [53]. Fluorescent tomographic imaging was conducted using a CLSM (FV-300; Olympus, Tokyo, Japan) with Ar 488 nm and He-Ne 543 nm lasers. Filters of 510–530 nm and ≥610 nm were utilized to detect SYTO 9 and propidium iodide, respectively [51,54]. A 100× oil-immersion objective lens was used. The assay was performed in triplicate for each material, including the control.

### 4.5. Viable and Total Cell Counting

The specimens were removed from the plug, gently rinsed twice with PBS, and transferred to an Eppendorf tube containing 2 mL of PBS after the incubation. To detach the biofilm from the dentin surface, the specimens were ultrasonicated for 5 min, vigorously shaken for 1 min, and ultrasonicated again for 5 min. Following biofilm detachment, the resulting suspension containing the detached biofilm cells was homogenized, serially diluted 10-fold, and plated onto Mitis Salivarius Agar. Viable cell or colony-forming unit counting was performed after anaerobic incubation for 48 h at 37 °C. The assay was performed in five replicates per treatment. 

For total cell counting, DNA was extracted using the NucleoSpin Microbial DNA Kit (Macherey-Nagel, Düren, Germany), following the manufacturer’s instructions. The extracted genomic DNA was quantified and stored at −20 °C until further processing. Quantitative analysis of the total bacterial count was performed using a modified Invader PLUS assay (BML, Inc., Tokyo, Japan), as previously described [51].

### 4.6. Adenosine Triphosphate Bioluminescence Assay

Following the biofilm detachment procedure, the number of viable bacteria remaining on the specimens was determined using the BacTiter-Glo™ microbial cell viability assay (Promega Corp., Madison, WI, USA). Luminescence was measured with a GloMax^®^ microplate reader (Promega Corp., Madison, WI, USA), following the protocol described in a previous study [55].

### 4.7. Acid Production Testing

After the detachment of the biofilm and incubation period, suspensions of *S. mutans* were homogenized, washed, and adjusted to an optical density of 0.05 at 600 nm. A volume of 100 μL of the bacterial suspension was added to the test solution of the CAT21 caries activity test (Morita Co., Tokyo, Japan) and incubated at 37 °C for 48 h. Acidogenic ability was assessed every 6 h over 48 h. Scoring was performed using a modified 7-point grading system with 0.5-point intervals based on a 4-point system [56].

### 4.8. EPMA

After 24 h of incubation, the specimens were embedded in a chemically polymerized resin (Nissin, Kanagawa, Japan). The resin block was halved using a diamond disk. The exposed surface was sequentially polished with 2400 grit and 4000 grit SiC paper (Marumoto Struers KK, Tokyo, Japan). Fluoride, silver, and zinc ions on the dentin surface were analyzed by elemental mapping using a wavelength-dispersive X-ray spectroscopy EPMA (EPMA1601) with an image observation function following established protocols [53,57]. A sample without any material or biofilm served as the negative control, whereas biofilms formed without any material served as positive controls.

### 4.9. Investigation of Gene Expression Related to Bacterial Adhesion

To study the transcription of genes involved in biofilm formation, biofilms of *S. mutans* formed on the specimens were collected and washed twice with PBS. The bacterial pellet was resuspended in TRI reagent (Molecular Research Center, Inc., Cincinnati, OH, USA) and mechanically disrupted using Lysing Matrix B in a MagNA Lyser at 7000 rpm for 30 s. RNA was isolated using a Direct-zol RNA kit (Zymo Research, Irvine, CA, USA) following previously described protocols [58]. RNA was reverse-transcribed using SuperScript VILO Master Mix (Thermo Fisher Scientific), and quantitative polymerase chain reaction with cDNA was conducted on a StepOnePlus real-time polymerase chain reaction system using the SYBR Green detection method. The 16S rRNA gene served as an internal control for data normalization. The primers used are listed in Table 2. The assay was performed for six replicates per treatment.

### 4.10. Statistical Analysis

Statistical analyses were performed using the GraphPad Prism 9 software (GraphPad Software, Inc., La Jolla, CA, USA). Data are expressed as the mean ± standard deviation, where appropriate. The Kruskal–Wallis test followed by a post hoc Dunn’s multiple comparison test were used to compare the test materials and their corresponding controls. The acid productions of each experimental group and their corresponding controls were compared using the Mann–Whitney U test. All tests were performed using a two-tailed significance threshold of *p* < 0.05.

## 5. Conclusions

Clinpro White Varnish (CP), which incorporated NaF and TCP, demonstrated the most effective antibiofilm activity. Its mechanisms effectively inhibited the bacterial adhesion and transcription of genes associated with biofilm formation. SDF, which contains silver and fluoride ions, reduced the number of live bacteria; however, other findings indicated notable disparities. The antibiofilm effect of CD, which contains zinc and fluoride ions, was limited. The inhibition of biofilm formation and reduction in tooth hypersensitivity may be accomplished using TCP and NaF in a synergistic combination.

## Figures and Tables

**Table 1:** Materials used in this study.

Desensitizer	Composition	Application Procedure	Manufacturer
Saforide (SDF)	Silver diamine fluoride (SDF); 38% SDF consists of silver and fluoride ions stabilized by ammonia	Clean and dry the dentin surface. Apply SDF using a microbrush or cotton pellet for at least 1 min. Allow it to air dry and avoid rinsing post-application. Protective measures include the isolation of soft tissues with petroleum jelly. Reapply biannually for optimal results.	Bee Brand Medico Dental Co., Ltd., Osaka, Japan Lot number: 104TA
Caredyne Shield (CD)	Fluoro-zinc-silicate glass	Clean the area and isolate with cotton rolls. Mix two equal proportions of solution A and B and apply to the dentin surface using a microbrush for 20 s. Rinse with water after application.	GC Corporation, Tokyo, Japan Lot number: 2210261
Clinpro White Varnish (CP)	5% sodium fluoride (NaF) and tricalcium phosphate (TCP)	Clean and dry the teeth. Apply the varnish using a brush or applicator directly onto the dentin. The product is saliva-activated, adhering to both dry and moist teeth.	3M Dental, St. Paul, MN, USA Lot number: NF27309

**Table 2:** Primers used for the quantitative reverse transcription-polymerase chain reaction analysis.

Gene Name	Nucleotide Sequence	Reference
*16s rRNA*	F: 5′-CCATGTGTAGCGGTGAAATGC-3′ R: 5′-TCATCGTTTACGGCGTGGAC-3′	[59]
*gtfB*	F: 5′-AGCCGAAAGTTGGTATCGTCC-3′ R: 5′-TGACGCTGTGTTTCTTGGCTC-3′	[59]
*gtfC*	F: 5′-TTCCGTCCCTTATTGATGACATG-3′ R: 5′-AATTGAAGCGGACTGGTTGCT-3′	[59]
*gtfD*	F:5′-ACAGCAGACAGCAGCCAAGA-3′ R: 5′-ACTGGGTTTGCTGCGTTTG-3′	[59]
*comD*	F: 5′-TTCCTGCAAACTCGATCATATAGG-3′ R: 5′-TGCCAGTTCTGACTTGTTTAGGC-3′	[59]
*comE*	F: 5′-TTCCTCTGATTGACCATTCTTCTG-3′ R: 5′-GAGTTTATGCCCCTCACTTTTCAG-3′	[59]
*luxS*	F: 5′-CCAGGGACATCTTTCCATGAGAT-3′ R: 5′-ACGGGATGATTGACTGTTCCC-3′	[59]
*aguD*	F: 5′-ATCCCGTGAGTGATAGTATTTG-3′ R: 5′-CAAGCCACCAACAAGTAAGG-3′	[60]
*atpD*	F: 5′-ACTGGGTTTGCTGCGTTTG-3′ R: 5′-CCAGGCGGTTCATTCATCTGAC-3′	[61]

## Data Availability

The datasets used in this study are available from the corresponding author upon request.
